# Physiological Responses, Exercise Intensity, and Enjoyment During Ring Fit Adventure in Adults With Chronic Diseases: Single-Arm, Pre-Post Experimental Study

**DOI:** 10.2196/92345

**Published:** 2026-10-19

**Authors:** Muhammad Jazimin Haron, Shiek Abdullah Ismail, Gilbert Tian Seng Yeo

**Affiliations:** 1 Rehabilitation Services, Khoo Teck Puat Hospital, 90 Yishun Central, Singapore, 768828, Singapore, 65 66023528 ext 3528; 2 Post-Acute & Community Care, Woodlands Hospital, Singapore, Singapore

**Keywords:** exergaming, active video games, exercise, exercise physiology, exercise intensity, gamification, Ring Fit Adventure, Nintendo Switch

## Abstract

**Background:**

Physical inactivity and insufficient muscle-strengthening exercise remain important public health concerns among adults with chronic diseases. Active video games may offer an accessible way to support exercise participation, but many may not provide sufficient exercise intensity to produce meaningful physiological responses. Ring Fit Adventure (RFA) is an active video game that combines aerobic and resistance-based exercise. However, little is known about its physiological demands among adults with chronic diseases.

**Objective:**

This pilot study aimed to examine the acute physiological responses, exercise intensity, perceived exertion, and enjoyment during RFA gameplay in adults with stable chronic diseases.

**Methods:**

A single-arm, pre-post experimental study was conducted among 20 adults aged 40 to 65 years with at least one chronic disease. Participants were recruited through convenience and snowball sampling from a hospital-based setting and public recruitment channels. After baseline assessment and familiarization, participants completed 2 stages of RFA gameplay. Heart rate (HR) was continuously monitored, while blood pressure (BP), capillary blood glucose (BG), and the rate of perceived exertion were measured at baseline and immediately after each stage. Enjoyment was assessed using the Exergame Enjoyment Questionnaire (EEQ).

**Results:**

All 20 participants completed the study, with no adverse events or missing data. The mean age was 54.3 (95% CI 51.25-57.25) years, and 10 participants were male. Hypertension was the most common condition (n=14, 70%), followed by hyperlipidemia (n=11, 55%), obesity (n=8, 40%), and diabetes (n=5, 25%). The mean continuous HR during gameplay was 111.6 (95% CI 102.6-120.6) bpm, corresponding to 67.2% of the age-predicted maximum HR, while peak HR reached 138.6 (95% CI 128.9-148.3) bpm, corresponding to 83.5% of the age-predicted maximum HR. HR increased significantly after World 1 by 16.7 (95% CI 11.42-21.88; *P*<.001) bpm and after World 2 by 20.7 (95% CI 13.28-28.12; *P*<.001) bpm. Rate of perceived exertion also increased significantly after World 1 by 2.83 (95% CI 2.32-3.33; *P*<.001) points and after World 2 by 3.75 (95% CI 3.18-4.32; *P*<.001) points. Capillary BG decreased after World 1 by 1.4 (95% CI −1.95 to 0.78; *P*<.001) mmol/L and after World 2 by 1.6 (95% CI −2.46 to −0.80; *P*=.01) mmol/L, with no hypoglycemic events. No significant changes were observed in systolic or diastolic BP. The mean enjoyment score was 77.6 out of 100 (95% CI 74.7-80.5).

**Conclusions:**

RFA elicited moderate average exercise intensity with periods of vigorous intensity, reductions in capillary BG, stable postexercise BP, and high enjoyment among adults with stable chronic diseases. These findings suggest that RFA may be a feasible and engaging home-based exercise option for this population. However, the small, heterogeneous sample, absence of a control condition, and single-session design limit generalizability. Larger controlled longitudinal studies are needed to determine its effects on exercise adherence, physical activity levels, and cardiometabolic outcomes.

## Introduction

Regular physical activity is a cornerstone in the prevention and management of noncommunicable diseases such as cardiovascular diseases, diabetes, and obesity [1]. The World Health Organization (WHO) [2] recommends that adults accumulate at least 150 to 300 minutes of moderate-intensity or 75 to 150 minutes of vigorous-intensity aerobic physical activity per week, in addition to muscle-strengthening exercises on 2 or more days per week. Despite well-established benefits, population-level physical activity has declined globally in recent decades, with approximately 1 in 3 adults worldwide classified as insufficiently active [3]. In Singapore, although overall physical activity prevalence is relatively high (84.7%), only 35.7% of residents reported engaging in sufficient muscle-strengthening exercise in 2024 [4]. This poses a significant public health concern, as physical inactivity is a major risk factor for noncommunicable diseases [5], while age-related and inactivity-related loss of muscle mass is associated with multimorbidity, functional decline, and reduced quality of life [6].

Individuals with noncommunicable diseases, especially cardiometabolic conditions like hypertension, type 2 diabetes, and obesity, are a key population for physical activity interventions. These individuals often face barriers to conventional exercise, including time constraints, low motivation, and limited access to structured exercise environments. For this population, improvements in cardiovascular fitness, glycemic control, and blood pressure (BP) are key therapeutic goals.

Home-based exercise is a promising strategy to address these challenges by offering greater accessibility, convenience, and flexibility, particularly for busy adults and individuals who are less comfortable exercising in the gym setting [7]. One such approach is the use of active video games (AVGs), which combine physical movement with entertainment and gameplay. These commercially available AVGs are increasingly popular and have demonstrated potential to increase physical activity levels [8]. Early examples include Dance Dance Revolution (Konami, Japan), Wii Fit (Nintendo Wii, Japan), and Just Dance, played across multiple platforms. However, many AVGs, including Wii Fit [9], are relatively low in exercise intensity and may not consistently meet recommended physical activity guidelines required for meaningful health benefits. Furthermore, a systematic review by Staiano and Flynn [10] analyzing 64 peer-reviewed studies reported that the majority of the studies on AVGs focused on balance-related outcomes, with none examining cardiovascular or cardiorespiratory parameters. This gap in the literature limits our understanding of whether AVGs can generate an adequate cardiovascular stimulus to improve cardiorespiratory fitness and metabolic health, which is central to chronic disease prevention and management.

An emerging AVG with the potential to deliver a comprehensive, multimodal exercise stimulus is Ring Fit Adventure (RFA)**,** developed by Nintendo for the Nintendo Switch console (Nintendo). RFA has 2 motion sensor controllers (Joy-Con): one secured to the player’s thigh via a leg strap to capture their lower-body movement and another controller integrated into a handheld Pilates ring (Ring-Con) that can detect upper-limb movement as well as pushing and pulling forces. In contrast to traditional AVGs, RFA integrates upper body and lower body movements, thereby providing both cardiovascular and resistance training. This distinct design positions RFA as a promising tool for individuals to engage in exercise at home, particularly for those seeking accessible and engaging alternatives to conventional exercise, as well as a way to incorporate resistance exercise, which is lacking among Singaporeans.

Despite its increasing popularity, limited research has been conducted to evaluate its effectiveness in meeting physical activity guidelines or its impact on key physiological markers such as BP and heart rate (HR). Existing studies have investigated the energy expenditure during RFA in children with overweight and obesity [11] and compared the energy expenditure during RFA using indirect calorimetry and an HR monitor [12]. Other studies examined the suitability of RFA exercise intensity for older adults [13] and changes in body composition among women [14] and compared RFA with traditional gym training [14]. There was also one study that evaluated the cardiovascular responses to a different AVG [15]. This highlights a lack of research directly assessing cardiovascular and metabolic responses specific to RFA, particularly in individuals with chronic diseases. Given the unique ability of RFA to incorporate both cardiovascular and resistance exercises, further investigation is needed to investigate its potential as a serious tool for exercise that meets the global recommendations, as well as in the prevention and management of noncommunicable diseases. Therefore, this single-session pilot study aimed to examine acute physiological responses, exercise intensity, and enjoyment during RFA gameplay in adults with selected stable chronic diseases. Specifically, we assessed changes in HR, BP, capillary blood glucose (BG), perceived exertion, and enjoyment after 2 stages of gameplay. We hypothesized that participants would achieve at least moderate exercise intensity during gameplay and report high enjoyment levels.

## Methods

### Study Recruitment

This was a single-arm, pre-post experimental study designed to examine acute physiological responses, exercise intensity, and enjoyment during RFA gameplay in adults with selected stable chronic diseases. Participants were recruited through convenience and snowball sampling between October 2023 and August 2024. Convenience sampling methods included referrals from health care professionals, posters at Khoo Teck Puat Hospital, Singapore, and an online research-focused public Telegram mobile application. Blinding was not implemented as participants and investigators were aware of the intervention during the gameplay. Sample size calculation was performed based on the mean and SD of a previous study [16], with *α*=.05 and power set as 0.8. Sample size of 15 was derived, and taking into consideration the possibility of participant dropouts, a target of 20 participants was set.

### Study Participants

Interested participants were screened for eligibility (Textbox 1) via email. The target age range was selected because it represented the age group with the highest prevalence of chronic disease [4]. The intention to include participants with any chronic disease and not differentiate them by specific chronic disease was to preserve ecological validity. In addition, these conditions were common among Singaporeans. Cardiovascular responses among people living with stable chronic diseases are also similar.

Textbox 1. **Study eligibility criteria.**

**Inclusion criteria**

- Adults aged 40 to 65 years with at least one chronic disease (hypertension, hyperlipidemia, diabetes, or obesity) and on medication
- Individuals deemed safe for exercise, defined as fulfilling at least one of the following criteria:
  - Has undergone medical screening and referred by doctor to this study or any exercise program or still has follow-up appointments
  - Has been exercising regularly for at least 30 minutes each session, 3 times a week at moderate intensity for the past 3 months without any signs and symptoms suggestive of cardiovascular disease
  - Has achieved the clinical targets for their respective chronic disease:
    - Participants with diabetes only: hemoglobin A_1C _≤8%
    - Participants with hypertension only: average systolic blood pressure ≤140/90 mm Hg
    - Participants with hyperlipidemia only: low-density lipoprotein (LDL) ≤4.1, high-density lipoprotein (HDL) ≥1.2, and triglycerides (TG) ≤1.7
    - Participants with hyperlipidemia, hypertension, and diabetes: LDL ≤2.6, HDL ≥1.2, and TG ≤1.7
    - Participants with hyperlipidemia and diabetes: LDL ≤2.6, HDL ≥1.2, and TG ≤1.7
    - Participants with hyperlipidemia and hypertension: LDL ≤3.4, HDL ≥1.2, and TG ≤1.7
- Participants who are fully vaccinated against COVID-19
- Participants who are able to ambulate independently
- Participants who are able to perform exercises in standing, including jogging on the spot
- Participants who are able to speak and understand English

**Exclusion criteria**

- Participants with history of cancer, cardiac disease, or pulmonary diseases
- Participants with musculoskeletal conditions that limit movement and exercise
- Participants who are frail and have poor balance
- Participants with symptoms suggestive of cardiovascular disease such as chest pain at rest and during exertion
- Participants with poor cognition and unable to follow multistep instructions
- Participants with pregnant

### Study Procedures

Participants who met the inclusion and exclusion criteria received information about the study in an information package and were informed of the session date. Participants were told to avoid vigorous activities for 24 hours prior to the session, to avoid caffeinated drinks, and to have a light meal 1 to 2 hours prior to their session. The timing and composition of the presession meal were not standardized or recorded.

After providing written informed consent and completing the International Physical Activity Questionnaire (IPAQ), anthropometric measurements were taken. Height was measured using the clinic stadiometer (Seca 286), while body composition was measured using a bioimpedance analyzer (InBody 770). Five minutes later, baseline parameters were measured while participants were seated; BP and HR were measured using a BP monitor (HEM-7322, Omron), and BG was measured using a glucometer (Accu-Chek Performa, Roche). Capillary BG was selected as a pragmatic acute physiological measure because the study aimed to examine immediate pre-post responses to a single gameplay session in a real-world exercise setting.

Participants were taught the modified rate of perceived exertion (RPE) scale (0 to 10) and were then asked to rate their RPE at rest. They then wore a HR monitor chest strap (Polar H10) synced to an iPad via Bluetooth and using the Polar Team app for monitoring. The Polar H10 HR monitor has been validated for accurate HR monitoring during exercise. In addition, participants wore a leg strap on the left thigh containing a wireless controller and held the Ring-Con, which incorporates another wireless controller, with both hands.

Participants then underwent a familiarization round for about 5 to 10 minutes at low intensity. After the familiarization round, participants were given 5 minutes of seated rest, and pre-exercise BP, HR, and RPE were measured. Once BP and HR had returned close to baseline, participants began playing the first stage of the adventure mode (World 1). Participants were encouraged to do their best and had to complete the game at their own pace. Time to complete was not controlled and was dependent on each individual’s effort. Throughout the game, HR was measured continuously via the HR chest strap and Polar Team app on iPad. Gameplay ceased if the participant experienced a fall, extreme shortness of breath, chest pain, dizziness, headache, or loss of consciousness.

Upon completion of “World 1,” BP, HR, RPE, and BG were measured immediately while seated. After 10 minutes of rest, participants moved on to the next stage of the game, entitled “World 2.” Upon completion of “World 2,” BP, HR, RPE, and BG were measured immediately while seated. Participants were given time to rest, and reassessments were done every 5 minutes until close to baseline and if indicated. The timing of the game was not controlled, and participants had to complete the game to the best of their ability. While resting, participants completed the Exergame Enjoyment Questionnaire (EEQ). The EEQ is a 20-item questionnaire for measuring how much players enjoyed an exergame. Questions were grouped into 4 categories: immersion, intrinsically rewarding activity, control, and exercise. Each subject’s responses were on a 5-point Likert scale: strongly disagree, disagree, neutral, agree, and strongly agree. Some questions were reverse scored. Participants received a shopping voucher worth SGD $20 (US $14.93) as a token of appreciation. A summary flowchart of the methods is shown in Figure 1 below.

**Figure 1.**
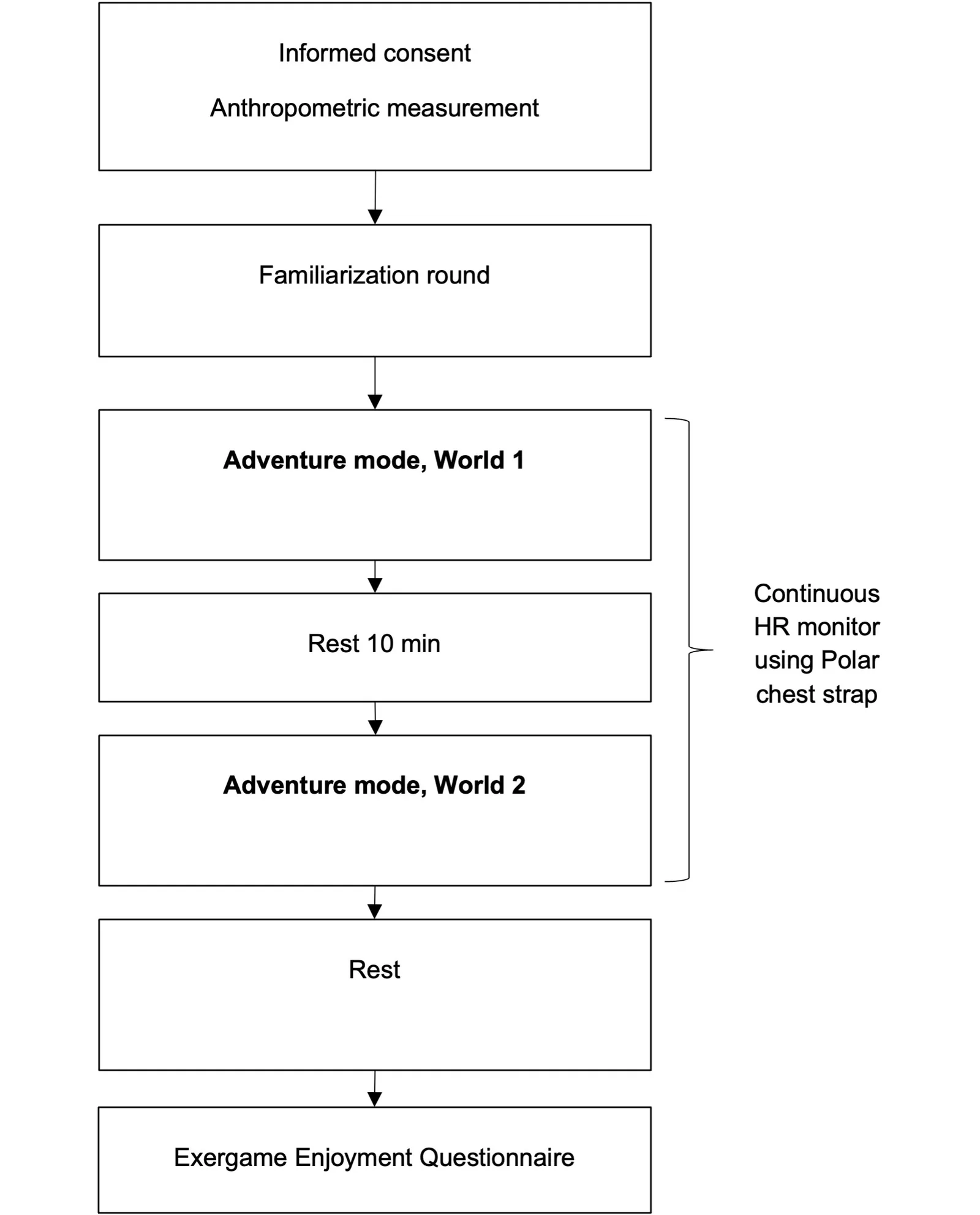
Study flowchart. HR: heart rate.

### Data Analysis

Responses were exported to Microsoft Excel, and data were analyzed using descriptive statistics and 2-tailed paired *t* tests in SPSS (IBM Corp) with significance level (*α*) set at .05. Descriptive statistics were used for participant characteristics, while 2-tailed paired *t* tests were used to compare baseline and postexercise physiological measures.

### Ethical Considerations

This study involving human participants was reviewed and approved by the National Healthcare Group (NHG) Domain Specific Review Board (DSRB), Board D, with reference number 2021/01064. All study procedures were conducted in accordance with the ethical standards of the institutional research committee. During the informed consent process, participants were given sufficient information and as much time as needed to decide on participation. This included information on the purpose of the study, study procedures, and potential risks. They were informed that participation is voluntary and that they have the autonomy to withdraw at any point of time, even during the exercise session, without the need to provide justification. Written informed consent was obtained, and a copy was given to all participants. The value of the token of appreciation was sufficient and not too large to coerce participation.

Privacy and confidentiality were strictly maintained, with all collected data deidentified and assigned study codes prior to analysis. No personally identifiable information was included in the dataset used for analysis or in any reports arising from this study. Study data were stored securely with password protection and were only accessible to members of the research team. There are no images or materials in this paper that contain identifiable information of participants. Therefore, no additional consent for publication of identifiable images was required.

## Results

### Participant Characteristics

Twenty participants (10 male and 10 female) met the inclusion criteria and were recruited. No participants dropped out during the study, and there were no adverse events. There were also no missing data. The average age was 54.3 (95% CI 51.3-57.3) years, and the most common chronic disease was hypertension (n=14, 70%). Almost half of the participants had hyperlipidemia (n=11, 55%) and obesity (n=8, 40%), while only 5 (25%) participants had diabetes. Table 1 shows the baseline characteristics of the participants.

Baseline BP and HR values were within expected resting ranges: systolic BP 128.1 (95% CI 120.9-135.2) mm Hg, diastolic BP 82.2 (95% CI 77.6-86.7) mm Hg, HR 71.1 (95% 65.6-76.5) bpm, and mean postprandial capillary BG 7.3 (95% CI 6.4-8.2) mmol/L. Changes observed in BP, HR, BG, and RPE for both Worlds are given in Tables 2 and 3.

**Table 1.** Participant characteristics (N=20).

Characteristics	Values
Age in years, mean (95% CI)	54.3 (51.3-57.3)
Sex, n (%)	
Male	10 (50)
Chronic diseases, n (%)	
Obesity (BMI>30 kg/m^2^)	8 (40)
Diabetes	5 (25)
Hypertension	14 (70)
Hyperlipidemia	11 (55)

**Table 2.** Blood pressure (BP), heart rate (HR), blood glucose (BG), and rate of perceived exertion (RPE) at baseline and after World 1.

Parameter	Baseline	World 1	Difference	*P* value
HR (bpm), mean (95% CI)	71.1 (65.6 to 76.5)	87.7 (80.2 to 95.2)	16.7 (11.42 to 21.88)	<.001
SBP^a^ (mm Hg), mean (95% CI)	128.1 (120.9 to 135.2)	130.8 (123.0 to 138.5)	2.70 (−3.40 to 8.80)	.40
DBP^b^ (mm Hg), mean (95% CI)	82.2 (77.6 to 86.7)	83.6 (78.3 to 88.8)	1.4 (−1.99 to 4.79)	.43
BG (mmol/L), mean (95% CI)	7.3 (6.4 to 8.2)	5.9 (5.4 to 6.4)	−1.4 (−1.95 to 0.78)	<.001
RPE, mean (95% CI)	0.25 (0.08 to 0.42)	3.08 (2.6 to 3.6)	2.83 (2.32 to 3.33)	<.001

a SBP: systolic blood pressure.

b DBP: diastolic blood pressure.

**Table 3.** Blood pressure (BP), heart rate (HR), blood glucose (BG), and rate of perceived exertion (RPE) at baseline and after World 2.

Parameter	Baseline	World 2	Difference	*P* value
HR (bpm), mean (95% CI)	71.1 (65.6 to 76.5)	91.8 (82.8 to 100.7)	20.7 (13.28 to 28.12)	<.001
SBP^a^ (mm Hg), mean (95% CI)	128.1 (120.9 to 135.2)	125.0 (117.4 to 132.5)	−3.10 (−10.1 to 3.91)	.40
DBP^b^ (mm Hg), mean (95% CI)	82.2 (77.6 to 86.7)	79.7 (74.7 to 84.6)	−2.5 (−6.10 to 1.10)	.19
BG (mmol/L), mean (95% CI)	7.3 (6.4 to 8.2)	5.7 (5.3 to 6.0)	−1.6 (−2.46 to −0.80)	.001
RPE, mean (95% CI)	0.25 (0.08 to 0.42)	4.0 (3.4 to 4.6)	3.75 (3.18 to 4.32)	<.001

a SBP: systolic blood pressure.

b DBP: diastolic blood pressure.

### HR Response

HR values were 87.7 (95% CI 80.2-95.2) bpm after World 1 and 91.8 (95% CI 82.8-100.7) bpm after World 2. When compared to baseline, these were significantly higher by 16.7 (95% CI 11.42-21.88; *P*<.001) bpm for World 1 and 20.7 (95% CI 13.28-28.12; *P*<.001) bpm for World 2. The average HR during the entire game (both Worlds), which was continuously recorded by the Polar Team app, was 111.6 (95% CI 102.6-120.6) bpm. The baseline HR was 71.1 (95% CI 65.6-76.5) bpm, with a difference of 40.6 (95% CI 33.9-47.2) bpm compared with the average HR. This was significantly higher than baseline (*P*<.001) and was about 67.2% of the age-predicted maximum HR, which is classified as moderate intensity. The average peak HR recorded was 138.6 (95% CI 128.9-148.3) bpm, which is about 83.5% of the age-predicted maximum HR and was classified as vigorous intensity. HR measured at the end of the Worlds appeared lower, as it was measured while participants were seated using the BP monitor and as the participant cooled down. The average exercise duration was 3 (4.1%) minutes in zone 5 (90%-100% HR max), 7 (9.65%) minutes in zone 4 (80%-90% HR max), 17 (24.2%) minutes in zone 3 (70%-80% HR max), 25 (33.8%) minutes in zone 2 (60%-70% HR max), and 20 (27.95%) minutes in zone 1 (<60% HR max).

### RPE Response

RPE values after completing both Worlds 1 and 2 were 3.08 (95% CI 2.6-3.6) and 4.00 (95% CI 3.4-4.6), which is classified as “moderate” to “somewhat hard.” When compared with baseline, these values were significantly higher by 2.83 (95% CI 2.32-3.33; *P*<.001) for World 1 and 3.75 (95% CI 3.18-4.32; *P*<.001) for World 2. This is consistent with the intensity level based on the HR response.

### BG Response

The average BG was 5.9 (95% CI 5.4-6.4) mmol/L after World 1 and 5.7 (95% CI 5.3-6.0) mmol/L after World 2. When compared with baseline, this was significantly lower by 1.4 (95% CI –1.95 to 0.78; *P*<.001) mmol/L for World 1 and 1.6 (95% CI –2.46 to –0.80; *P*=.01) mmol/L for World 2. There were no hypoglycemic episodes. When comparing participants with and without diabetes, there was a larger decrease in BG after World 1 and World 2 compared with baseline for those with diabetes. This may be due to a higher pre-exercise BG for those with diabetes (mean 8.7, SD 2.9 mmol/L) compared to those without diabetes (mean 6.8, SD 1.6 mmol/L).

### BP Response

When compared with baseline, there were no significant changes in systolic BP and diastolic BP after completing World 1 and World 2. BP was measured while participants were seated using a BP monitor and would probably capture a slightly lower BP than during gameplay.

### Enjoyment Level

Using the EEQ, where higher scores indicate greater enjoyment, the mean score was 77.6 (95% CI 74.7-80.5) out of a maximum of 100 points.

## Discussion

### Enjoyment Levels of RFA

This pilot study provides preliminary evidence that RFA can elicit moderate average exercise intensity, periods of vigorous intensity, and high enjoyment among adults with selected stable chronic diseases during a single supervised gameplay session. The key findings include a mean exercise intensity of 67.2% of the age-predicted maximum HR during gameplay, significant improvements in BG levels, and high enjoyment scores, all achieved without adverse events.

### Exercise Intensity of and Physiological Responses to RFA

The HR responses observed during RFA gameplay indicate that participants achieved exercise intensities consistent with current physical activity recommendations. The average continuous HR of 67.2% of the age-predicted maximum HR places RFA firmly within the moderate-intensity exercise category as defined by the American College of Sports Medicine. More importantly, peak HRs reaching 83.5% of the maximum suggest that RFA can also provide vigorous-intensity exercise periods, offering the potential for comprehensive cardiovascular training.

The RPE scores of 3.08 to 4.00, corresponding to “moderate” to “somewhat hard” intensity, corroborate the objective HR measurements and suggest that participants’ subjective experience aligns with the physiological demands of the exercise. This concordance between objective and subjective measures of exercise intensity is crucial for exercise prescription and monitoring in clinical populations.

The significant reduction in capillary BG after both gaming sessions, by approximately 1.4 to 1.6 mmol/L, suggests that RFA may have acute glucose-lowering effects. This response is physiologically plausible, as contracting skeletal muscle increases glucose uptake during exercise. Participants with diabetes appeared to show larger reductions, suggesting potential relevance for this group. Importantly, no hypoglycemic episodes were observed, indicating that the exercise intensity was well tolerated in this single-session setting. However, these findings should be interpreted cautiously. Presession meal timing and composition were not standardized, and there was no nonexercise control condition. Therefore, the observed changes should be regarded as preliminary acute responses rather than evidence of sustained glycemic benefit.

### Safety Profile and Clinical Implications

The absence of adverse events and the maintenance of stable BP throughout the study period underscore RFA’s safety profile in adults with chronic diseases. The lack of significant BP changes post exercise suggests that RFA does not induce excessive cardiovascular stress that could be concerning for individuals with hypertension or other cardiovascular risk factors.

This safety profile is particularly important given that 70% (14/20) of participants had hypertension and many had multiple comorbidities. The ability to achieve moderate-to-vigorous intensity exercise without adverse cardiovascular responses supports RFA’s potential as a safe exercise modality for clinical populations who may have limited access to supervised exercise programs.

### Enjoyment and Adherence Potential

The high enjoyment scores (a mean of 77.6, 95% CI 74.7-80.5, out of 100) on the EEQ represent a significant finding for long-term exercise adherence. Traditional exercise programs often suffer from poor adherence rates, particularly in individuals with chronic diseases who may face additional barriers to physical activity. The gamification elements of RFA, including character progression, virtual rewards, and immersive gameplay, appear to successfully enhance the exercise experience beyond traditional forms of physical activity.

This level of enjoyment is crucial for sustainable behavior change, as intrinsic motivation and enjoyment are strong predictors of long-term exercise adherence [17]. The combination of effective exercise intensity and high enjoyment levels positions RFA as a potentially valuable tool for addressing physical inactivity.

### Comparison With Prior Research

Our findings extend the limited existing research on RFA’s physiological effects. While Comeras-Chueca [11] examined energy expenditure in children with overweight and obesity, our study is among the first to comprehensively evaluate cardiovascular responses, BG effects, and subjective experiences in adults with chronic diseases. The exercise intensities we observed are comparable to those reported for other AVGs, but RFA’s ability to consistently achieve moderate-to-vigorous intensity levels distinguishes it from many commercially available AVGs that typically elicit only light-to-moderate intensity exercise.

The glucose-lowering effects we observed align with established research on acute exercise responses but demonstrate that these benefits can be achieved through gamified exercise in a home setting. This is particularly relevant given the growing body of evidence supporting the role of regular physical activity in chronic disease management.

### Clinical Applications and Public Health Implications

From a clinical and public health perspective, these findings suggest that exergaming platforms such as RFA could serve as a valuable adjunct to conventional exercise prescriptions. In particular, RFA may serve as a valuable adjunct to traditional exercise prescriptions, particularly for individuals who face barriers to conventional exercise programs. The home-based nature of RFA addresses common obstacles such as transportation difficulties, time constraints, and facility access, while the engaging format may appeal to individuals who find traditional exercise monotonous or intimidating.

These findings also contribute to the growing field of digital health and exergaming by highlighting the potential of gamified exercise platforms to deliver both cardiovascular and resistance training. For health care providers, RFA represents a potential tool for exercise prescription in a format that patients may find more acceptable and sustainable than traditional exercise recommendations. The objective monitoring capabilities of modern gaming systems could also facilitate remote exercise monitoring and progression tracking.

### Limitations

Several limitations should be acknowledged. Participants had heterogeneous chronic diseases, including hypertension, hyperlipidemia, diabetes, and obesity, with some having multimorbidity. These conditions may have some influence on HR, BP, and glucose responses during exercise. However, the small sample size limited subgroup analyses and adjustments.

The single-session design precluded the assessment of long-term adherence, sustained engagement, exercise progression, and longer-term outcomes such as fitness, body composition, BP control, or glycemic regulation. The absence of a control condition also means that the observed changes, particularly in BG, cannot be attributed solely to gameplay.

Capillary BG was used as a pragmatic acute measure, but interpretation was limited by the lack of standardization of presession meal timing and composition. Therefore, glucose reductions may reflect both exercise-related uptake and expected postprandial decline.

Finally, participants were recruited through convenience and snowball sampling from a limited setting and were screened and found to be safe for exercise. This may limit generalizability to broader populations, including older adults and those with more severe or unstable chronic diseases.

### Future Research Directions

Longitudinal studies are needed to evaluate the sustained effects of regular RFA gameplay on cardiovascular fitness, metabolic parameters, and chronic disease management. Research examining optimal dosing strategies and integration with traditional exercise programs would inform clinical implementation.

Investigation of RFA’s effects in specific clinical populations, such as individuals with diabetes requiring insulin management or those with more severe cardiovascular disease, would expand the evidence base for clinical applications. Additionally, studies comparing RFA to traditional exercise modalities in terms of both physiological outcomes and long-term adherence would provide valuable comparative effectiveness data.

The potential for RFA to serve as a gateway to increased overall physical activity, rather than a replacement for traditional exercise, represents an important area for future investigation. Understanding how gamified exercise experiences might influence broader physical activity behaviors could inform public health strategies for addressing physical inactivity.

### Conclusions

In this single-session pilot study, RFA elicited moderate average exercise intensity, with intermittent periods of vigorous intensity, among adults with selected stable chronic diseases. The game combines cardiovascular and resistance-based exercise, both of which are important for chronic disease prevention and management. Gameplay appeared feasible, engaging, and well tolerated in participants with stable conditions such as hypertension, hyperlipidemia, diabetes, and obesity. No adverse events were observed, and there were no clinically concerning spikes in BP or BG levels. The gamified design of RFA may make exercise more enjoyable by allowing users to progress, level up, and defeat enemies through physical activity. This may improve motivation and support exercise adherence, which is important for long-term chronic disease management. However, the findings remain preliminary because of the small heterogeneous sample, a lack of a control group, and absence of longitudinal follow-up. Larger controlled studies are needed to evaluate the long-term effects of RFA on exercise adherence, physical activity levels, body composition, fitness, cardiometabolic outcomes, and chronic disease control.

### Implications

RFA can be considered as exercise and be used as part of chronic disease management for patients with hypertension, hyperlipidemia, diabetes, and obesity. It caters to users with different fitness levels, as the game allows calibration through a few questions and both upper and lower limb movement tests. It has many different game modes, including the adventure mode that is designed to gradually challenge the user at each stage. Game developers should continue to incorporate game elements that can elicit physiological responses sufficient for improving health.

## Abbreviations

AVG: active video game
BG: blood glucose
BP: blood pressure
DSRB: Domain Specific Review Board
EEQ: Exergame Enjoyment Questionnaire
HR: heart rate
IPAQ: International Physical Activity Questionnaire
NHG: National Healthcare Group
RFA: Ring Fit Adventure
RPE: rate of perceived exertion
WHO: World Health Organization
